# Optimization of a Low Cost and Broadly Sensitive Genotyping Assay for HIV-1 Drug Resistance Surveillance and Monitoring in Resource-Limited Settings

**DOI:** 10.1371/journal.pone.0028184

**Published:** 2011-11-23

**Authors:** Zhiyong Zhou, Nick Wagar, Joshua R. DeVos, Erin Rottinghaus, Karidia Diallo, Duc B. Nguyen, Orji Bassey, Richard Ugbena, Nellie Wadonda-Kabondo, Michelle S. McConnell, Isaac Zulu, Benson Chilima, John Nkengasong, Chunfu Yang

**Affiliations:** 1 Division of Global HIV/AIDS, Center for Global Health, Centers for Disease Control and Prevention (CDC), Atlanta, Georgia, United States of America; 2 Department of Health and Human Services/US CDC, Hanoi, Vietnam; 3 CDC/GAP, Abuja, Nigeria; 4 Community Health Sciences Unit, Malawi Ministry of Health, Lilongwe, Malawi; 5 Thailand Ministry of Public Health/US CDC Collaboration, Nonthaburi, Thailand; 6 Global AIDS Program CDC-Zambia, Lusaka, Zambia; Centro Nacional de Microbiología - Instituto de Salud Carlos III, Spain

## Abstract

Commercially available HIV-1 drug resistance (HIVDR) genotyping assays are expensive and have limitations in detecting non-B subtypes and circulating recombinant forms that are co-circulating in resource-limited settings (RLS). This study aimed to optimize a low cost and broadly sensitive in-house assay in detecting HIVDR mutations in the protease (PR) and reverse transcriptase (RT) regions of *pol* gene. The overall plasma genotyping sensitivity was 95.8% (N = 96). Compared to the original in-house assay and two commercially available genotyping systems, TRUGENE® and ViroSeq®, the optimized in-house assay showed a nucleotide sequence concordance of 99.3%, 99.6% and 99.1%, respectively. The optimized in-house assay was more sensitive in detecting mixture bases than the original in-house (N = 87, P<0.001) and TRUGENE® and ViroSeq® assays. When the optimized in-house assay was applied to genotype samples collected for HIVDR surveys (N = 230), all 72 (100%) plasma and 69 (95.8%) of the matched dried blood spots (DBS) in the Vietnam transmitted HIVDR survey were genotyped and nucleotide sequence concordance was 98.8%; Testing of treatment-experienced patient plasmas with viral load (VL) ≥ and <3 log10 copies/ml from the Nigeria and Malawi surveys yielded 100% (N = 46) and 78.6% (N = 14) genotyping rates, respectively. Furthermore, all 18 matched DBS stored at room temperature from the Nigeria survey were genotyped. Phylogenetic analysis of the 236 sequences revealed that 43.6% were CRF01_AE, 25.9% subtype C, 13.1% CRF02_AG, 5.1% subtype G, 4.2% subtype B, 2.5% subtype A, 2.1% each subtype F and unclassifiable, 0.4% each CRF06_CPX, CRF07_BC and CRF09_CPX.

**Conclusions:**

The optimized in-house assay is broadly sensitive in genotyping HIV-1 group M viral strains and more sensitive than the original in-house, TRUGENE® and ViroSeq® in detecting mixed viral populations. The broad sensitivity and substantial reagent cost saving make this assay more accessible for RLS where HIVDR surveillance is recommended to minimize the development and transmission of HIVDR.

## Introduction

Treatment of HIV-1 infection with highly active antiretroviral therapy (HAART) in the past decades has remarkably reduced HIV/AIDS related mortality and morbidity. However, the emergence of drug resistance in persons on antiretroviral therapy (ART) and the transmission of drug-resistant HIV strains to newly infected persons are a major threat to the global success on HIV prevention and treatment effort [1], [2], [3]. Recent years, under multilateral supports for HIV treatment and prevention programs, especially the U.S. President Emergency Plan for AIDS Relief (PEPFAR) with the targets of treating two million HIV-infected people with ART, preventing five million new HIV infection and care for 10 million HIV-infected people and AIDS orphans, access to antiretroviral drugs (ARVs) has been scaled up rapidly in resource-limited countries where availability of laboratory monitoring is often limited or lacking [4], [5]. This creates the potential for HIV drug resistance (HIVDR) emergence and transmission in these settings. Detection and monitoring of HIVDR by molecular genotyping is pivotal to ensure ongoing regimen efficacy. It is the standard of care in resource-rich countries [2], [6]; however in resource-limited countries, HIVDR testing is not generally available or it is too costly to be used in routine monitoring of patients receiving ARVs. Therefore, the World Health Organization (WHO) recommends population-based surveillance and monitoring of HIVDR in resource-limited settings [2], [4], [7]. Pattern and rates of transmitted and acquired drug resistant HIV variants will collectively inform regional and global recommendations on which ARVs to maintain or change in first and second-line regimens [7].

Population sequencing-based genotyping methods including ViroSeq®, TRUGENE® and in-house assays are widely used, and the most informative and affordable genotyping methods for monitoring patients on ART in clinical practice [8], [9], [10], [11]. However, ViroSeq® and TRUGENE®, the two FDA-approved genotyping assays were designed for HIV-1 group M subtype B viruses which are the predominant HIV-1 strains in resource-rich countries. In addition, these commercial kits are expensive and less sensitive to non-B subtypes, limiting their utility in resource-limited settings [12], [13], [14]. There have been no commercially available HIV-1 genotyping assays designed for non-B subtypes and circulating recombinant forms (CRFs) that are predominant viral strains in resource-limited countries. Moreover, the demand for low cost and sensitive genotyping methods is increasing with the establishment and expansion of laboratory molecular monitoring in these settings [15], [16].

The most frequently used HIVDR genotypic assays are assays that detect resistance mutations in the reverse-transcriptase (RT) and protease (PR) genes [17], [18], [19]. The minimal genotyping requirements for these two regions are PR codons 10–99 and RT codons 41–240 [3], [20], [21]. Our original in-house assay [22] has its limitations: (1). It does not cover the entire PR gene region required for resistance testing; (2). For some HIV-1 subtypes or CRFs, some sequencing primers generate higher background noises which would affect the detection of mixture bases. We redesigned two primers for RT-PCR, one for nested PCR and four sequencing primers in the optimized assay.

In-house assays are relatively inexpensive and sensitive for multiple subtypes, but in-house assays should only be implemented after adequate validation, including evaluating assay's performance with various HIV-1 subtypes and CRFs [3], [20], [23]. Factors that could contribute to genotyping quality include type of assay/kit used, specimen handling and storage, level of experience of technicians performing the analysis, heterozygosity of sequences, and viral subtypes in clinical samples [11], [21]. In this study, therefore, we validated sensitivity, accuracy and specificity of the optimized in-house assay. We also evaluated the assay applicability to HIVDR surveillance and monitoring.

## Materials and Methods

### Samples

A total of 381 samples were used in this study: 151 samples were used for validation and 230 for application. For validation, we included 111 HIV-1 positive plasma and 10 dried blood spot (DBS) samples, as well as 30 HIV-1 negative DBS samples. For application, we tested 132 plasma and 98 DBS samples. The detailed information on these samples is described in Table 1.

**Table 1:** Summary of samples used in the study, including plasma and dried blood spots (DBS).

Origin	No. of samples	Type of sample	Collection year	ART status	Median VL _log10_ (range)	Storage condition	VL measurement	
**Samples For validation (N = 151)**								
Cameroon	38	Plasma	2007	Experienced	4.05 (2.60–5.57)	−70°C	Roche Amplicor v1.5	
Thailand	31	Plasma	2006	Naïve	4.65 (3.14–5.58)	−70°C	Roche COBAS TaqMan	
Zambia	27	Plasma	2006–2007	Experienced	4.26 (3.34–5.88)	−70°C	Roche Amplicor v1.5	
	30^a^	DBS	2005–2006	Not applicable	Not applicable	−70°C	Not applicable	
PT panels	15	Plasma	2009–2010	NA^d^	4.13 (3.93–4.75)	−70°C	NA	
	10	DBS	2010	NA	3.78 (3.23–4.29)	−70°C or 5 DBS shipped at ambient	NA	
**Samples for application (N = 230)**								
Vietnam	72	Plasma	2007–2008	Naïve	Not done	−70°C	Not done	
	72^b^	DBS	2007–2008	Naïve	Not done	−70°C	Not done	
Malawi	34	Plasma	2009	Experienced	4.07 (2.25–5.89)	−70°C	Abbott m2000rt	
Nigeria	26	Plasma	2009	Experienced	4.02 (2.18–6.41)	−70°C	BioMerieux EasyQ	
	26^c^	DBS	2009	Experienced	3.97 (2.18–5.64)	Room temperature for an average 85 days	BioMerieux EasyQ	

a HIV negative specimens collected from pregnant women in Tanzania used for assay specificity analysis; ^b^ Plasma-matched DBS samples collected from voluntary counseling and testing (VCT) sites in Ho Chi Minh City enrolled in an HIV-1 threshold survey; ^c^Plasma-matched DBS samples collected from patients enrolled in the Nigeria HIVDR perspective monitoring survey at 12-15 months after commencement of first line antiretroviral therapy; ^d^ Not available.

### Dried blood spot sample preparation and storage

Dried blood spot samples were prepared by spotting 100 µl of whole blood onto each of the five preprinted circles on a Whatman 903 filter paper (Whatman Inc, Piscataway, NJ) and were then dried overnight at ambient temperature. The next day, a piece of glassine paper was folded and a DBS card was placed in the folded paper, and 10–20 wrapped DBS cards were then packaged in a Bitran bag containing desiccant sacks and a humidity indicator card and sealed. The packaged DBS cards were stored either at −70°C for Vietnam samples or at room temperature for an average of 85.31±42.66 days (median 83.5 days) for Nigeria samples. The DBS cards were shipped to the WHO Specialized Drug Resistance Laboratory at the Centers for Disease Control and Prevention (CDC) (Atlanta, GA, USA) either on dry ice for the samples from Vietnam or at ambient temperature for the DBS samples from Nigeria. All samples were stored at −70°C upon the arrival at CDC.

### Viral RNA and total nucleic acid extraction

The QIAamp mini-viral RNA kit (Qiagen, Valencia, CA) was used to extract RNA from all plasma samples for validation purpose. Details for viral load (VL) measurement on samples from Cameroon, Thailand and Zambia were described previously [22], [24], [25]. For Malawi samples, Abbott m2000 automatic sample preparation system (0.2 ml extraction protocol) was used to extract the plasma RNA. For Nigeria and Vietnam plasma and DBS samples, the NucliSens® EasyMAG^TM^ automatic sample preparation system (BioMérieux, Durbam, NC) was used to extract the plasma RNA and blood total nucleic acid (TNA). To extract TNA, one DBS spot was cut out per specimen and placed in a 2ml of NucliSENS® lysis buffer (Biomeriuex, Durham, NC) for 30 min at room temperature with gentle rotation. Nucleic acid was then extracted from DBS samples using the NucliSENS® EasyMag® automated extraction system following the manufacturer's instructions. Nucleic acid was eluted in 25 µl of NucliSENS® Extraction Buffer 3 and stored at −80°C until use.

### RT-PCR and nested PCR

Sequences of RT-PCR and sequencing primers that were re-designed or modified based on the original assay [22] and HIV-1 sequences available at the Los Alamos HIV Database (www.hiv.lanl.gov) are shown in Table 2. Three RT-PCR and four sequencing primers were replaced during the optimization process. All primers used were synthesized at CDC Biotechnology Core Facility. Two oligonucleotides that mixed at a ratio of 1:1 (w/w) were used as the forward primer for one step RT-PCR. One-step RT-PCR was performed in a 50 µl reaction, which consisted of 10 µl of RNA or TNA extracts, 0.16 µM each of primers PRTM-F1 and RT-R1, and 0.5 µl SuperScript^TM^ III one step RT/Platinum® *Taq* high Fidelity Enzyme Mix and 1x reaction buffer mixture containing Mg2+ and eoxyribonucleotide triphosphates (dNTPs) (Invitrogen, Carlsbad, CA). RT-PCR condition was an initial cycle RT step at 50°C for 45 min and 94°C for 2 min, and followed by 40 cycles of PCR at 94°C for 15 sec, 50°C for 20 sec, 72°C for 2 min and an extension at 72°C for 10 min. For nested PCR, 2 µl of RT-PCR product was added to a 50 µl reaction containing 0.12 µM of each of the inner primers PRT-F2 and RT-R2, 1x GeneAmp Gold Buffer II, 2 mM MgCl_2_, 400 µM each dNTP and 2.5 U of AmpliTaq Gold LD DNA polymerase (Applied Biosystems, Foster City, CA). After initial denaturation at 94°C for 4 min, 40 cycles of PCR were performed in GeneAmp 9700 thermocycler with the PCR conditions as 94°C for 15 sec, 55°C for 20 sec and 72 for 2 min and following an extension at 72°C for 10 min. In the case of the failed first RT-PCR attempt, PRTM2-F1 was used as rescue primer to replace PRTM-F1 to account for mutations occurring within the primer binding site. The nested PCR product was confirmed by 1% agarose gel electrophoresis with a product size of 1,084 base pairs. The confirmed PCR products were purified using Exo-SAP IT PCR purification kits and used for cycle sequencing reaction with BigDye terminator cycle sequencing kit 3.1 (Applied Biosystems, CA).

**Table 2:** Primers used in the optimized in-house assay.

Primer name	Sequence (5′→3′)	Location(based on HXB2)	Purpose
PRTM-F1*	F1a-TGAARGAITGYACTGARAGRCAGGCTAATF1b-ACTGARAGRCAGGCTAATTTTTTAG	2057–20852068–2092	RT-PCR, one of mixture componentsRT-PCR, one of mixture components
PRTM2-F1	TAGGGA RAATYTGGCCTTCC	2090–2109	Rescue RT-PCR primer
RT-R1	ATCCCTGCATAAATCTGACTTGC	3370–3348	RT-PCR
PRT-F2	CTTTARCTTCCCTCARATCACTCT	2243–2266	Nested PCR & sequencing
RT-R2	CTTCTGTATGTCATTGACAGTCC	3326–3304	Nested PCR & sequencing
SeqF3	AGTCCTATTGARACTGTRCCAG	2556–2577	Sequencing
SeqR3	TTTYTCTTCTGTCAATGGCCA	2639–2619	Sequencing
SeqF4	CAGTACTGGATGTGGGRGAYG	2869–2889	Sequencing
SeqR4	TACTAGGTATGGTAAATGCAGT	2952–2931	Sequencing

*: PRTM-F1 is a mixture of primers F1a and F1b at a ratio of 1:1 (w/w).

### Sequence analysis

DNA sequencing of HIV-1 *pol* was performed in 3730 DNA genetic analyzer (Applied Biosystems). Six sequencing primers overlapping the entire amplicon were used (Table 2). Sequencing raw data were edited with ChromasPro, v1.5 (Technelysium Pty Ltd, Australia) and confirmed by a second technician. To double check for all mixed bases, we also used a web-based sequence analysis tool, ReCall [26], in which minor peak calling was set at 15% of the main peak. To rule out PCR contamination, phylogenetic analyses were performed on all newly obtained sequences by MEGA 4 [27]. Sequence quality was also checked by Stanford HIVdb program. Sequences with frame shifts or stop codons were excluded from analysis. For transmitted HIVDR surveillance, WHO surveillance drug resistance mutation (SDRM) list was used [28]. For HIVDR monitoring surveys, drug resistance-associated mutations in PR and RT were interpreted using the Stanford Genotypic Resistance Interpretation Algorithm (http://hivdb.stanford.edu/pages/algs/HIVdb.html). Pairwise nucleotide sequence identity and discrepancy were analyzed using BioEdit [29].

### Sensitivity, accuracy and specificity of the assay

As for HIV-1 drug resistance genotyping, there are no standardized or reference method (gold standard) to evaluate analytic and clinical performance in molecular genotyping for HIV-1 group M viruses. We validated the new method according to WHO/HIVResNet drug resistance guidelines [21], including participation in an external quality assessment (EQA) program, proficiency testing (PT) panels and comparing the results between new method and the original method already established in our laboratory [22].

Because the validation criteria were difficult to define based on the complexity of samples tested in this multi-subtype evaluation and all currently available assays (commercial or in-house) were unable to genotype 100% of the samples tested [16], for this study we used the genotyping sensitivity intervals as ≥95% for samples with VL ≥3 log copies/ml; accuracy was defined as detection of 99% of known DR mutation codons, and reproducibility/precision was defined as ≥98% nucleotide identities in ≥ 90% of pairwise comparisons. In this study, sensitivity and reproducibility of the assay were assessed by comparing the current genotyping results from 96 field collected samples of known VL with those of the original assay [22] from Cameroon, Zambia and Thailand. The assay was also evaluated using TRUGENE® system GL12 with plasma from DigitalPT (N = 5), a HIVDR PT program offered by AccuTest at Boston, MA, USA and using ViroSeq® system v 2.8 with plasma from Virology Quality Assurance program (VQA, N = 5), a WHO-sponsored HIIVDR PT program and offered by VQA at Chicago, IL, USA. Additionally, the precision of the assay was evaluated using 4 replicates of a second VQA plasma PT panel (N = 5) and 3 of 4 replicates were tested with the optimized in-house assay and the remaining one was tested with TRUGENE®. The precision test was performed by 3 technicians. In addition, 10 DBS panels shipped at two different temperature conditions from VQA were also tested by the optimized assay. Specificity was determined by testing 30 HIV-negative DBS specimens collected from pregnant women in Tanzania.

### Applying the assay for surveillance of transmitted HIVDR and HIVDR prevention monitoring surveys in resource-limited countries

Seventy two matched plasma and DBS samples from newly HIV-diagnosed persons in Vietnam were tested. For HIVDR monitoring surveys, we applied the optimized assay for resistance testing in samples collected from patients 12–15 months after the commencement of ART in two monitoring surveys. For the Malawi monitoring survey, 34 plasma samples from patients with VL ranged from 2.25 to 5.89 log10 copies/ml were tested. In Nigeria monitoring survey, 26 matched plasma and DBS samples with plasma VL ranged from 2.18 to 6.41 log10 copies/ml were analyzed.

### HIV-1 subtyping

HIV-1 subtyping for the newly obtained sequences was performed using the REGA 4 HIV-1 Genotyping Tool [30]. Phylogenetic analyses were further conducted using neighbor-joining method included in the MEGA 4 for sequences with unclassifiable subtypes. Reference sequences were obtained from the Los Alamos HIV Database (www.hiv.lanl.gov). Sequences obtained in the study were submitted to GenBank and their accession numbers are JN885633 to JN885719.

### Reagent cost comparison

To estimate reagent cost savings by using the optimized broad sensitive genotyping assay, we calculated reagent cost per test of the assay and compared it with the reagent costs of commercially available genotyping systems, TRUGENE® and ViroSeq®. We used current U.S. market values in dollars for all the reagents we used in the in-house assay including RNA/TNA extraction, RT-PCR, nested PCR, PCR amplification confirmation, PCR purification and sequencing reactions. These reagent cost estimates did not include the cost for running test controls and any repetitions when needed.

### Statistical analysis

Wilcoxon Signed-Rank test was used to analyze the difference in number of nucleotide mixtures detected between the optimized and original in-house assays. The statistical significance was considered when P value was <0.05.

### Ethical consideration

In accordance with United States regulations and international guidelines, the CDC human subjects review process determined this activity to be non-research and the protocol was approved by the Associate Director for Science (ADS), National Center for HIV, Hepatitis, STD and TB Prevention, CDC, Atlanta. All the study protocols were approved by local institutional review boards: the National Health Sciences Research Committee, Ministry of Health of Malawi; the National Institute for Medical Research at Lagos, Nigeria; the Thailand Ministry of Public Health and Sirriraj Hospital, Mahidol University, Thailand; the University of Alabama, Birmingham and the University of Zambia Research Ethics Committee, Zambia.

## Results

### Validation of the optimized in-house assay

#### Sensitivity

The sensitivity of the optimized in-house assay was evaluated with 96 HIV-1 positive plasma samples collected from Cameroon, Thailand and Zambia. Of these, all 5 samples with VL <3 log10 copies/ml and 87 (95.6%) of 91 samples with VL ≥3 log10 copies/ml were genotyped, resulting in an overall genotyping rate of 95.8% (92/96) comparing to 96.8% (93/96) by the original assay.

#### Accuracy

The accuracy of the optimized assay was first assessed by comparing 87 paired nucleotide sequences generated by the original in-house assay [22] and the optimized assay using ReCall and BioEdit programs. The mean nucleotide identity was 99.3±0.50% (mean ± SD) among paired nucleotide sequences. Wilcoxon signed-rank test was used to compare original and optimized in-house assays in basecalling for mixed bases and revealed that the optimized assay detected significantly more mixed bases than the original one (P<0.001). However, this difference did not translate into differences in HIVDR mutations. Among 144 DR mutations detected in paired samples, we did not detect any complete discordant mutations at DR mutation sites and only 11 partially discordant DR mutation sites including 3 in PR and 8 in RT (Table 3) were seen. The overall DR codon agreement was 99.8% between the 87 paired samples.

**Table 3:** Discordant drug resistance-associated amino acid positions in protease and reverse transcriptase from 87 plasma samples^a^ genotyped by the original and optimized in-house assays.

Amino acid position	Mutation	Amino acid detected in the original assay(No. of sample)	Amino acid detected in the newly optimized assay	No. partially discordant mutation
Protease32	V32A	V (87)	V (86), AV (1)	1
33	L33F	L (84), F (3)	L (84), F (3)	0
35	E35G	G (86), EG (1)	G (85), EG (2)	1
71	A71V	A (86), V (1)	A (86), AV (1)	1
74	T74S	T (82), S (5)	T (82), S (5)	0
Reverse Transcriptase62	A62V	A (86), AV (1)	A (86), AV (1)	0
65	K65R	K (86), R (1)	K (86), KR (1)	1
67	D67N	D (86), DN (1)	D (86), DN (1)	0
69	T69S/N	T (82), ST (1), N (1), NT (3)	T (82), ST (1), N (1), NT (3)	0
90	V90I	V (85), I (1), IV (1)	V (85), IV (2)	1
98	A98G	A (86), G (1)	A (86), G (1)	0
101	K101E/Q	K (84), E (2), Q (1)	K (84), E (2), Q (1)	0
103	K103N	K (77), N (8), KN (2)	K (76), N (8), KN (3)	1
106	V106A/I	V (82), A (1), I (3), IV (1)	V (83), A (1), I (3)	1
118	V118I	V (83), I (3), IV (1)	V (83), I (3), IV (1)	0
138	E138A	E (86), A (1)	E (86), A (1)	0
179	V179D/T	V (82), D (3), DV (1), T (1)	V (82), D (3), DV (1), T (1)	0
181	Y181C	Y (81), C (3), CY (3),	Y(81), C (3), CY (3)	0
184	M184V	M (78), V (8), IMV (1)	M (78), V (8), IMV (1)	0
188	Y188C/L	Y (85), L (1), CY (1)	Y (86), L (1)	1
190	G190A	G (84), A (3)	G (83), A (2), AG (2)	1
210	L210F	L (86), F (1)	L (86), F (1)	0
215	T215A	T (86), AT (1)	T (87)	1
221	H221Y	H (87)	H (86), HY (1)	1

a : Five samples that did not generate full-length sequences for protease (codon 13 to 99) and reverse transcriptase (codon 1 to 251) were excluded for the analysis. Among them, 4 sequences were generated by the original assay and 1 by the optimized assay.

**Figure 1:**
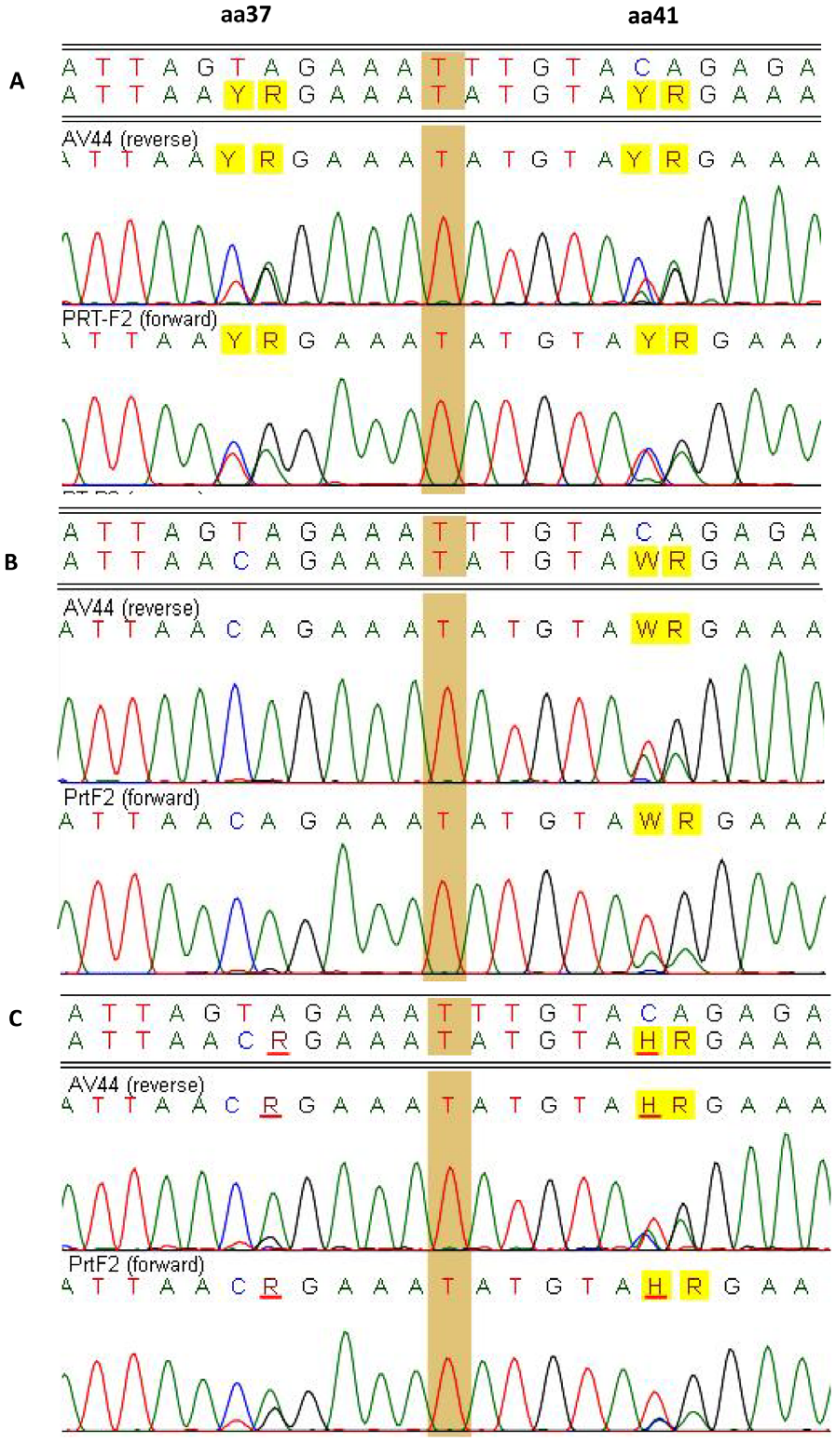
Difference of mixture chromatographs generated independently by 3 different operators using the optimized in-house assay from one PT sample. Panel A shows 2 codons (37 and 41 of RT) with nucleotide base calling of AYR; Panel B shows the AWR at codon 41 (the second peaks at codon 37 were not detected in this replicate); Panel C shows ACR at codon 37 (minor T was not called by the ReCall at the cutoff of 15%) and AHR at codon 41 (almost equal height of second and third peak at the 2^nd^ position).

Testing 10 plasma PT panel samples (five from DigitalPT and five from VQA) using the optimized in-house and TRUGENE® or ViroSeq® assays also indicated that the optimized in-house assay appeared to detect more mixed bases than commercial kits. However, DR mutation site differences only occurred in 2 of 76 DR mutations between the optimized in-house and TRUGENE® and 2 of 44 between the in-house and ViroSeq® in mixed bases. The overall sequence identity was 99.6±0.41% between the in-house and TRUGENE® (Table 4), and 99.1±0.65% between the in-house and ViroSeq® (Table 5). Further examination to see whether more sensitive detection of base mixtures in the optimized in-house assay is a reproducible event, we analyzed 4 replicates of 5 samples that were tested by three optimized in-house assay runs and one TRUGENE® run under different operators. We found highly concordant sequence identities ranging from 98.22% to 99.65%. The minor differences observed in sequence identity were caused by base mixtures (Table 6). For example, at codons 37 and 41 of RT in sample 3 (Fig 1), one replicate detected mixture RY at the 2^nd^ and 3^rd^ positions of codon 37, and the second replicate did not find any mixtures, while the third replicate showed lower, yet visible, second peaks comparing to the first replicate. Similarly at the 2^nd^ position of codon 41, the first replicate revealed a Y (C/T), the second replicate showed a W (A/T), and the third replicate revealed an H (A/C/T) while TRUGENE® replicate detected a Y (C/T). Nucleotide mixtures also caused some mismatched DR mutations between the replicates. For instance, one in-house replicate missed 3 mixed codons (K65KR, D67DN and T69IT) in sample 2 among the 4 replicates. However, the minor peaks of nucleotide bases at these three codons could be seen, but were below the mixture cutoff (15%) on the chromatogram by ReCall. Thus these mixtures were not counted and resulted in the codon discrepancy. Another partial discordant example was the DR mutation M184MV, which was detected in sample 5 by all 3 in-house replicates but not found in TRUGENE® replicate. These results indicated that the sequences generated by population-based sequencing were highly reproducible but the sensitivity at detecting low frequency of drug resistant HIV variants was very challenging.

**Table 4:** Pairwise sequence identity analysis between the optimized in-house and TRUGENE® assays.

DigitalPT panel	Optimized in-house vs TRUGENE
No. of sample	5
% Nucleotide identity	99.6±0.40
Mean nucleotide mixture	11.4 vs 6.2
% amino acid identity	98.9±0.48
No. of DR mutation	76 vs 74
Partial discordant mutation (%)	2 (2.6)

**Table 5:** Pairwise sequence identity analysis between the optimized in-house and ViroSeq® assays.

VQA PT panel	Optimized in-house vs ViroSeq®
No. of sample	5
% Nucleotide identity	99.1±0.65
Mean nucleotide mixture	26.4 vs 18.8
% amino acid identity	97.5±1.75
No. of DR mutation	44 vs 42
Partial discordant mutation (%)	2 (4.5)

**Table 6:** Genotyping reproducibility of replicate PCR products generated from independent RT-PCR amplification process by 3 different operators in a 5-member proficiency testing panel received from VQA.

				No. of drug resistance mutations				
Sample ID	HIV-1 VL (log10)	HIV-1 Subtype	% Nucleotide sequence identity	Replicate Tests				No. Partially discordant mutation
				IH1*	IH2	IH3	TG^#^	
1	3.76	B	98.83±0.18	1	1	1	1	0
2	4.13	C	99.65±0.23	10	10	7	10	3
3	4.19	F	98.22±0.30	0	0	0	0	0
4	3.93	B	99.08±0.11	5	5	5	5	0
5	4.75	C	99.58±0.08	6	6	5	4	3

*IH1-3: tests were independently performed by 3 operators using the optimized in-house assay; ^#^:TRUGENE® assay.

#### Sensitivity on DBS samples

DBS samples are recommended by WHO for HIVDR surveillance in resource-limited settings in treatment-naïve populations [21]. The mission of our laboratory at CDC is to support HIVDR surveillance in PEPFAR-supported countries; thus, we evaluated the assay sensitivity with two matched DBS experimental panels shipped under different temperature conditions from VQA. The optimized in-house assay was able to genotype all 5 DBS samples shipped with dry ice and 4 of 5 DBS samples shipped at ambient temperature with DBS VL ranging from 3.17 to 3.98 log10 copies/ml. The failed sample GEN001BS.04C was the sample with the lowest VL of 3.17 log10 copies/ml (f 7).

**Table 7:** Genotyping efficiency and drug resistance-associated mutations identified in protease (PR) and reverse transcriptase (RT) by the optimized in-house assay from dried blood spots (DBS) PT panels.

Panel sample ID	Shipping conditions	Plasma VL (Log10)	DBS VL (Log10)		RT-PCR result	Subtype	Drug resistance mutation PR RT	
DBS panel A								
GEN001BS.01A	Dry ice	3.78	3.51	+		F	None	None
GEN001BS.02A	Dry ice	3.73	3.76	+		B	None	M184MV, K103N
GEN001BS.03A	Dry ice	4.29	3.98	+		C	None	M41L, K103N, M184V, T215Y
GEN001BS.04A	Dry ice	3.23	3.17	+		B	L10I, L23I, L33F, M46L, I54V, A71T, V82A, N88G, L90M	M41L, E44D, A62V, D67N, L74V, L100I, K103N, L210W, T215Y, H221Y
GEN001BS.05A	Dry ice	3.87	3.80	+		B	None	K103N, Y181C, P225H
DBS panel C								
GEN001BS.01C	Ambient	3.78	3.51	+		F	None	None
GEN001BS.02C	Ambient	3.73	3.76	+		B	None	M184MV, K103N
GEN001BS.03C	Ambient	4.29	3.98	+		C	None	M41L, K103N, Y181CY, M184MV, T215Y
GEN001BS.04C	Ambient	3.23	3.17			N/A*	N/A	N/A
GEN001BS.05C	Ambient	3.87	3.80	+		B	None	K103N, Y181CY, P225H

*N/A:not available; bold and underlined residues were partially discordant resistance mutations from paired DBS shipped under different temperature conditions.

To evaluate the specificity of the optimized in-house assay**,** we tested HIV negative DBS samples (N = 30) collected from women attending ANC clinics in Tanzania and they were all found to be negative, resulting in the assay specificity of 100%.

EQA assessment results: Based on WHO/HIVResNet requirement to pass the PT panels, a drug resistance mutation (DRM) site score and nucleotide (nt) alignment score with consensus sequence of at least 99% (considering all 5 samples) must be achieved. The optimized in-house assay passed two sets of plasma PT panels with 100% DRM, 99.98% nt and 100% DRM, 99.88% nt scores, respectively. The assay showed high sequence concordance with the laboratories participating in the EQA program.

### Application of the assay in the surveillance of HIVDR in resource-poor countries

With the satisfactory validation results of the optimized in-house assay, we applied this assay in the surveillance of HIVDR in three PEPFAR-supported countries.

#### Threshold survey of transmitted HIVDR in recently HIV-infected population in Vietnam

For this survey, we tested 72 plasma and matched DBS specimens collected from individuals attending voluntary counseling and testing (VCT) in Ho Chi Minh City. We were able to genotype all 72 plasma and 69 (95.8%) DBS samples and sequence identity analysis (N = 69) indicated that overall nucleotide identity was 98.9%±0.62% between matched plasma and DBS samples. The sequence differences were caused by partially discordant mixture bases located in the three HIVDR codons in the RT region.

#### Detection of HIVDR development in ART-experienced patients from Malawi and Nigeria

We next utilized the optimized in-house assay in detecting HIVDR development in patients treated with first-line ARVs for 12-15 months in two monitoring surveys conducted in Malawi (N = 34) and Nigeria (N = 26). Genotyping was successful for all 46 plasma samples collected from virologically failed patients defined as VL ≥3 log10 copies/ml according to the WHO definition [4]. For patients with VL between 2.18 and <3 log10 copies/ml, 78.6% (11/14) plasma samples (7/8 from Nigeria and 4/6 from Malawi) were also successful. Furthermore, all 18 matched DBS samples from virologically failing patients and 4 of the 8 DBS samples with VL between 2.18 and <3 log10 copies/ml from Nigeria were genotyped. The nucleotide sequence identity between the 22 plasma and DBS pairs was 98.8±0.80%. For DR mutations, 90.4% DR mutations identified in plasma were also found in DBS. For the 9.6% discordant DR mutations identified, majority of them was partially discordant (7.5%) and only 2.1% were completely discordant.

### HIV -1 subtypes

Phylogenetic analyses revealed that the overall subtype distributions among the 236 newly obtained sequences were 43.6% CRF 01_AE, 25.6% C, 13.1% CRF02_AG, 5.1% G, 4.2% B, 2.5% A, 2.1% F, 2.1% unclassified (UC), and 0.4% each CRF06_CPX, CRF09_CPX and CRF-07_BC. Subtype distributions are different from country to country. For instance, all samples tested from Vietnam and Malawi were CRF-01_AE and subtype C, respectively, while multiple subtypes were identified from samples collected from Cameroon, Canada and Nigeria (Table 8).

**Table 8:** HIV-1 subtypes and circulating recombinant forms (CRFs) from samples genotyped by the optimized in-house assay.

Sample source	No. of Sample	A	B	C	F	G	CRF01_AE	CRF02_AG	CRF06_CPX	CRF07_BC		CRF09_CPX	UC
Cameroon	31	3			2	2		21				1	2
Malawi	32			32									
Nigeria	25	1				10		10	1				3
Zambia	25	1		24									
Thailand	31						31						
Vietnam	72						72						
Canada and US	20	1	10	5	3					1			
**Total**	**236**	**6**	**10**	**61**	**5**	**12**	**103**	**31**	**1**	**1**		**1**	**5**
**% subtype**	**100**	**2.54**	**4.24**	**25.85**	**2.12**	**5.09**	**43.64**	**13.14**	**0.42**	**0.42**		**0.42**	**2.12**

### Reagent cost comparison

Using the current U.S. market values in dollars for all the reagents used in the optimized in-house assay, we estimated that the reagent cost per test for the optimized in-house assay was $40.00, comparing to $213.20 for TRUGENE® and $172.86 for ViroSeq®. In the reagent cost calculations, we did not include the cost for assay controls and any need for repetition of tests, which would increase the cost of reagents for all the assays compared here.

## Discussion

The newly optimized in-house assay was broadly sensitive in genotyping multiple HIV-1 group M subtypes and CRFs from plasma and DBS collected from 6 resource-limited countries. The original in-house assay, although a success from the broad sensitivity perspective, was in need for improvement due to the concern of incomplete genotyping of PR gene and suboptimal sequence quality due to primer design [22]. The validation of the re-designed RT-PCR and some of the sequencing primers in the current study confirmed that the newly optimized in-house assay is comparable to the original in-house assay in assay sensitivity and specificity and it is also broadly sensitive to all group M subtypes and CRFs circulating in PEPFAR-supported countries. Pairwise nucleotide sequence identity analyses from sequences generated by the optimized in-house assay and the ones obtained from the original in-house assay and two commercially available genotyping systems indicated the optimized in-house assay produced comparable genotyping results. More importantly, the optimized in-house assay expanded genotyping codon coverage to include all PR mutations and improved sequence quality by reducing background noises to minimal, resulting in more sensitive mixture calling. The ability to detect nucleotide mixtures (low frequency viral strains) is important as recent studies have demonstrated that low frequency variants can grow rapidly and become predominant viral population under the selective drug pressure and lead to treatment failure [31], [32].

Given that the newly optimized in-house assay was broadly sensitive in genotyping B and non-B subtype viral strains of HIV-1 group M viruses, it would be expected that the assay would efficiently genotype plasma and DBS samples from various geographical areas, and this was verified by applying the assay in genotyping samples collected from patients enrolled in two perspective HIVDR monitoring surveys. The optimized in-house assay was able to genotype 100% of plasma samples collected from virological failure patients, defined as VL ≥3 log10 copies/ml [4] in the present study and over three-fourths of the patients with VL between 2.18 and <3 log 10 copies/ml. More importantly, genotyping was successful for all the 18 matched DBS samples collected from Nigerian patients with virological failure. It is worthy to note that these DBS samples had been stored at room temperature for an average of 85 days before shipping to our laboratory for testing. In addition, testing of two DBS PT panels shipped frozen or at ambient temperature revealed that genotyping was successful for all DBS samples except one with the lowest VL and shipped at ambient temperature. These results indicate that the optimized in-house assay is highly sensitive in genotyping both plasma and DBS samples. It is important to point out that interpretation of the genotyping results from the DBS samples stored at room temperature for a long period of time however, needs to be cautious since these DBS cards were packaged correctly and stored in an air-conditioning room with low humidity. These package and storage condition might have limited the true impact of suboptimal storage conditions existed in resource-limited settings on the quality of DBS cards and resulted in better genotyping efficiency. Studies have shown that correct packaging and storage of DBS are critical elements in ensuring successful genotyping results [33], [34], [35], [36]. In fact, our own data here generated from two identical DBS PT panels and shipped at frozen or ambient temperature also indicate that even overnight exposure of DBS samples with low VL to ambient temperature in domestic shipment could have some detrimental impact on DBS quality for genotyping. Comparing the performance of the optimized in-house assay with TRUGENE®, ViroSeq® and the original in-house assay, high nucleotide sequence identity was revealed; however, minor differences existed in mixture base callings. The optimized in-house assay detected more mixed bases than the commercial kits and our original in-house assay. Many factors could contribute to the sequence discordances at the mixed nucleotide sites in HIV genotyping including viral quasispecies, primer binding preference and location, Taq polymerase mis-incorporation, sequence quality, basecalling criteria or technical errors [11], [37], [38]. Because HIV-1 viruses are rapidly evolving quasispecies [39], there are multiple HIV-1 variants in one patient [37], [40]. Sequence identity and codon concordance are challenging when mixed bases are present [41]. It has been reported that ViroSeq® detected more mixtures (78%) than an in-house assay (22%) [10]. In contrast, our optimized in-house assay detected more mixture bases than other assays. This may be due to the fact that the optimized assay produced sequence chromatographs containing minimal background noise. To confirm this, we performed sequence editing for all validation samples (N = 102) including PT panels with ReCall program [26] using minor peak default mixture calling setting at >15% of the major peak in bi-directional sequences. We also independently tested one set of the PT panels by 3 different operators. These analyses showed that the optimized in-house assay gave more sensitive mixture calling. The variability in detecting nucleotide mixtures was likely due to the first-round RT-PCR [42] in sampling of quasispecies strains rather than by technical errors in the sequencing process [43]. The use of wide-spectrum degenerate primers and a mixture of two forward primers at slightly different binding sites in the initial run of RT-PCR are likely contributed to more mixtures calling in the optimized in-house assay. To what extent and by what factors the mixture variants could be affected and detected in HIV genotyping is a matter of speculation, which needs further studies.

Phylogenetic analyses indicated that the optimized in-house assay could genotype HIV-1 group M subtypes A (A1, A2), B, C, F (F1, F2), G and CRFs including CRF01_AE, CRF02_AG, CRF06_CPX, CRF07_BC, CRF09_CPX and UC with an overall sensitivity of 96% using specimens from different geographical regions around the world. Due to the limited availability of HIV-positive samples, we only genotyped a small number of subtype A and F viral strains and further studies are needed to confirm our findings with larger sample sizes on these viral strains. It has been reported that genotyping sensitivity with two FDA-approved systems using non-B subtypes varies [44]. Some studies indicated that these two systems performed well for B and non-B subtypes [9], [45], [46], [47] while others demonstrated that they were less sensitive to non-B subtypes and CRFs [12], [13], [14], [48]. For instance, only 52% of serum samples were genotyped in an Ethiopian threshold survey using ViroSeq® and TRUGENE® methods sequentially [49]. For genotyping DBS samples collected from subtype B infected persons, one study reported 78.8% genotyping rate by TRUGENE® [50] while another study reported 57.5% genotyping rate using ViroSeq® for DBS samples stored for one year at 4°C [51]. An additional study reported an even lower DBS genotyping rate of 38.6% by ViroSeq® system [40]. However, a study using ViroSeq® documented 100% genotyping rate for DBS samples collected from subtype B-infected patients with VL great than 2,000 copies/ml and 54.5% genotyping rate with DBS samples from patients with VL less than 2,000 copies/ml when DBS samples were stored at optimal conditions [52]. Thus, commercial genotyping systems might work well with DBS samples collected from subtype-B-infected patients. Genotyping of DBS samples using these genotyping systems in non-B subtypes needs to be further studied. In comparison to our original in-house assay, our own experience using these two commercial assays with non-B subtypes was also not satisfactory. They often required repetition of RT-PCR or sequencing due to failure to amplify or sequence in TRUGENE® and ViroSeq® assays [12], [14]. Compared to these commercial assays, the optimized in-house assay was not only sensitive, but also inexpensive. The assay could reduce the cost for genotyping reagents by 75%. The availability of low cost and broadly sensitive genotyping assay for plasma and DBS would make HIVDR surveillance and monitoring in resource-limited settings more accessible.

In conclusion, we have validated and improved a broadly sensitive and less expensive in-house genotyping assay for HIVDR surveillance and monitoring in resource-limited countries. Validation analyses indicate that the optimized in-house assay detected more mixed HIV-1 population than our original in-house assay and commercial genotyping kits. Given the high efficiency in genotyping diverse HIV-1 group M viral strains from plasma and DBS samples and substantial reagent cost saving, the optimized in-house assay could be applicable to DR genotyping in both ART-naive and -experienced populations according to current WHO recommendations for surveillance purpose [4].
